# Towards access for all: 1st Working Group Report for the Global Gene Therapy Initiative (GGTI)

**DOI:** 10.1038/s41434-021-00284-4

**Published:** 2021-09-08

**Authors:** Jennifer E. Adair, Lindsay Androski, Lois Bayigga, Deus Bazira, Eugene Brandon, Lynda Dee, Steven Deeks, Mohamed Draz, Karine Dubé, Mark Dybul, Umut Gurkan, Evelyn Harlow, Cissy Kityo, Michael Louella, Punam Malik, Vikram Mathews, Adrian McKemey, Henry Mugerwa, Daniel Muyanja, Olabimpe Olayiwola, Rimas J. Orentas, Alex Popovski, Jeff Sheehy, Francis Ssali, Moses Supercharger Nsubuga, John F. Tisdale, Els Verhoeyen, Boro Dropulić

**Affiliations:** 1 Fred Hutchinson Cancer Research Center, Seattle, WA USA; 2 University of Washington, Seattle, WA USA; 3 Roivant Social Ventures, New York, NY USA; 4 Joint Clinical Research Centre, Kampala, Uganda; 5 Georgetown University Medical Center, Washington, District of Columbia USA; 6 IQVIA, Durham, NC USA; 7 AIDS Action Baltimore, Baltimore, MD USA; 8 amFAR Institute for HIV Cure Community Advisory Board, New York, NY USA; 9 Delaney AIDS Research Enterprise Community Advisory Board, San Francisco, CA USA; 10 Martin Delaney Collaboratory Community Advisory Board, Bethesda, MD USA; 11 University of California at San Francisco, San Francisco, CA USA; 12 Case Western Reserve University, Cleveland, OH USA; 13 University of North Carolina, Chapel Hill, NC USA; 14 Ministry of Health, Kampala, Uganda; 15 Cincinnati Children’s Medical Hospital, Cincinnati, OH USA; 16 Christian Medical College, Vellore, India; 17 Caring Cross, Gaithersburg, MD USA; 18 Seattle Children’s Research Institute, Seattle, WA USA; 19 National Heart, Lung and Blood Institute, National Institutes of Health, Bethesda, MD USA; 20 Université Côte d’Azur, INSERM, C3M, Nice, France; 21 CIRI, INSERM, Université Lyon, CNRS, ENS de Lyon, Lyon, France

**Keywords:** Gene delivery, Haematological diseases

## Abstract

The gene and cell therapy field saw its first approved treatments in Europe in 2012 and the United States in 2017 and is projected to be at least a $10B USD industry by 2025. Despite this success, a massive gap exists between the companies, clinics, and researchers developing these therapeutic approaches, and their availability to the patients who need them. The unacceptable reality is a geographic exclusion of low-and middle-income countries (LMIC) in gene therapy development and ultimately the provision of gene therapies to patients in LMIC. This is particularly relevant for gene therapies to treat human immunodeficiency virus infection and hemoglobinopathies, global health crises impacting tens of millions of people primarily located in LMIC. Bridging this divide will require research, clinical and regulatory infrastructural development, capacity-building, training, an approval pathway and community adoption for success and sustainable affordability. In 2020, the Global Gene Therapy Initiative was formed to tackle the barriers to LMIC inclusion in gene therapy development. This working group includes diverse stakeholders from all sectors and has set a goal of introducing two gene therapy Phase I clinical trials in two LMIC, Uganda and India, by 2024. Here we report on progress to date for this initiative.

## Introduction

After three decades of research and clinical evaluation, the last 5 years in the gene and cell therapy field has witnessed the approval of multiple gene therapies for inherited and malignant diseases receive for drug status in Europe and the United States (U.S.). With hundreds of gene therapy clinical trials currently in progress for nearly as many diseases, the approval pipeline is expected to grow exponentially in the coming decade [1]. Dr. Scott Gottlieb, U.S. Food and Drug Administration (FDA) Commissioner from 2017 to 2019, predicted that as many as 10–20 new gene therapies could be approved per year by 2025, and the Massachusetts Institute of Technology predicts as many as 40 gene therapies approved by 2030 [2]. Indeed, 206 investigational new drug applications for gene therapy products were submitted to the FDA in 2018, an increase of 94% from the previous year. Unsurprisingly, financing in the regenerative medicine sector, which includes gene therapies, has seen an exponential increase from $6B U.S. in 2019, to $19.9 B U.S. in 2020 [3].

Much of the success of gene therapies is due to the focus of this approach on fundamental disease biology. Many gene therapies have the potential to constitute one-time treatments with lifelong curative potential, which is transformative for diseases where standard of care is limited in efficacy. This raises great hope for patients everywhere, but also concerns about how these treatments will be made available. Patients are not alone in these concerns, as researchers, clinicians, regulators, nonprofit entities including foundations and scientific societies, as well as for-profit and government entities including health systems have vested interests in the widespread success of gene therapy as a treatment strategy.

**Fig. 1:**
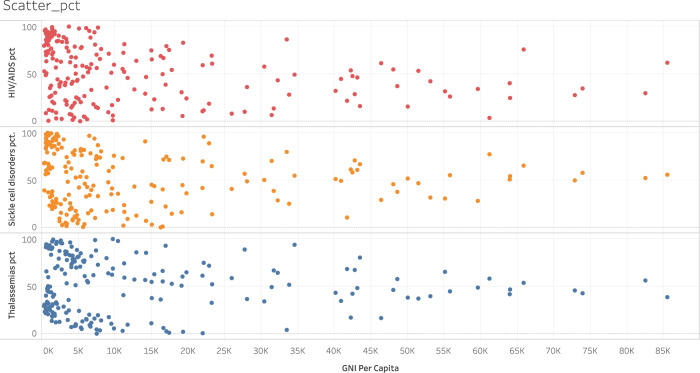
The highest prevalence of HIV/AIDS, sickle cell disease and thalassemia, and thus the populations who could most benefit from one-time curative treatments, are low- and middle-income countries. Each dot represents a single country. For each disease indication (y-axis), the prevalence of the disease is represented as patient count percentile (pct; rate metric per 100,000), as measured by the Institute for Health Metrics & Evaluation’s 2019 Global Health Data Exchange, and is plotted against the gross national income (GNI) per capita for the same country as reported by the World Bank 2021 classifications (x-axis).

At the forefront of reservations are the widely acknowledged high costs of approved gene therapy treatments, which range from $373 K U.S. to $2.1 M U.S. While these price tags reflect the incredible efforts undertaken by gene therapy pioneers to achieve initial approvals, they are not sustainable for developed countries, let alone low- and middle-income countries (LMIC), defined by the World Bank as those nations with a gross national income of less than or equal to $12,535 USD per capita. This affordability gap underscores the need for more cost-effective strategies to deliver gene therapy to make curative treatments accessible to all who need them. The gap comes into sharper view when considering two global health burdens for which multiple gene therapy candidates are already in clinical trials on the pathway to approval: human immunodeficiency virus (HIV) infection and hemoglobinopathies such as sickle cell disease and beta-thalassemia (Fig. 1). While the vast majority of the tens of millions of people living with HIV or a hemoglobinopathy reside in LMIC– 90% and 99%, respectively [4, 5] [https://AIDSinfo.unaids.org]– an overwhelming majority of gene therapy clinical trials are conducted in high-income countries such as the U.S. A https://clinicaltrials.gov query for interventional clinical trials with the search term “gene therapy” retrieves a total of 849 trials, while only six include sites in southeast Asia, and only four include sites in Africa. Sites in southeast Asia include India, where three trials for Gaucher’s disease, inherited retinitis or non-small cell lung cancer are registered, and Thailand, where three trials, two for beta-thalassemia and one for metastatic prostate cancer are registered. In Africa, all trials focus on hemophilia or cancer and all are registered in South Africa. Such an approach to research and development raises a significant question: will a gene therapy developed, tested, validated and approved in patients who do not represent the majority of cases for a given disease readily translate to those communities that bear the brunt of disease burden and thus could most benefit? Here the very underlying diversity in our human biology could undercut the most significant benefit of gene therapies, namely their direct focus on disease biology, if it does not consider the natural variation in populations where these diseases are most prevalent. Thus, early consideration of variations in biology for various patient populations should be prioritized in the context of gene therapy clinical trials and associated research. One example of biological variation is genetic, as Africa is known to be the most genetically diverse continent, with a recent deep genome sequencing study finding more than 3 million novel gene variants in 426 individuals from 50 ethnolinguistic groups [6]. This becomes highly relevant when gene editing efforts expand to these populations. Another aspect of variation to consider is disease variation. For instance, sickle cell disease claims the lives of many children in sub-Saharan Africa before diagnosis owing to limitations in diagnostic testing [7]. For HIV, two recent studies in Rakai, Uganda demonstrate latently-infected cells in persons living with HIV on antiretroviral drug therapy to be as much as threefold lower than compared to a similar patient cohort in the U.S. [8, 9].

As reviewed by Carvalho and colleagues [10], while the complexities of launching a gene therapy clinical trials in LMIC are diverse, there is a compelling need to accelerate progress at this critical time of growth of the field. The most frequently discussed barrier is affordability, with cost of therapy and reimbursement being most critical. Current timing in the field presents a significant opportunity to make transformative advances in affordability, as innovations required to implement gene therapy in LMIC will lower costs for these treatments and increase competition, which will in turn benefit patients everywhere. Importantly, these advances will also empower LMIC where these patients reside to find a place in the gene therapy value chain as vital stakeholders in the research and development ecosystem.

Initially, it was a simple phone call from Dr. Kityo of the Joint Clinical Research Center in Kampala, Uganda to Dr. Adair of the Fred Hutchinson Research Center, which revealed this shared vision. This interaction was quickly extended to Drs. Boro Dropulić and Rimas Orentas, who had previously founded a nonprofit called Caring Cross, whose mission is to enable affordable and sustainable access of cell and gene therapies, particularly to underserved populations around the world. It was quickly realized that our individual networks, if combined, could make transformative advances. To this end, the Global Gene Therapy Initiative (GGTI) was formed in the Fall of 2020, with an ambitious goal of launching a Phase I clinical trial for gene therapy to treat hemoglobinopathy or HIV infection in two LMIC by 2024. Here we report on the progress of this working group alliance to date and extend an open invitation to stakeholders everywhere to join our efforts.

### An alliance forms

The GGTI is an alliance of key stakeholders including clinicians, scientists, engineers, advocates, and community members including patients and caregivers brought together by the shared interest of enabling access and implementation of gene therapies as curative medicines for presently incurable diseases in LMIC. The impetus for group formation was multi-faceted, but relied heavily on cold-calls between individual stakeholders who immediately identified overlapping interests. These entities initiated and supported networking between individuals, institutions and continents which allowed parallel but disconnected efforts toward the same goal to form a collective for discussion and the sharing of ideas. From this, an organic alliance formed to catalyze the development and implementation of gene therapies for inherited and infectious diseases that disproportionately impact LMIC by advocating for appropriate research, clinical development, capacity-building, training, community acceptance, regulatory pathway approval, and sustainability.

### Where to begin

Early partners in the GGTI self-identified as key LMIC clinics where gene therapies could be implemented, and expressed a willingness to map out gaps in infrastructure and differences in regulatory approval processes, as well as barriers to patient and community acceptance. Because of our initial focus on hemoglobinopathies and HIV infection, it was important for initial clinical trial sites to be centers currently treating patients in high-prevalence regions. International GGTI stakeholders represent LMIC on two different continents and with different capacities for gene therapy implementation. Importantly, a unifying characteristic of both centers is the capacity for future deployment of gene therapies in their representative countries, India and Uganda respectively, as well as other LMIC in their continental regions.

The Christian Medical College (CMC) in Vellore, India, represents a clinical research center and regional training center of excellence with bone marrow transplant service capable of performing 200 transplants per year and the capacity for manufacturing blood cell gene therapy products locally [11, 12]. Half of the world’s beta-thalassemia carriers and affected births reside in South-East Asia. Moreover, while India as a nation is considered to have a low prevalence of HIV infection, as within other nations categorized as “low prevalence” there is substantial heterogeneity with sub-national pockets with higher prevalence. In India, these pockets are localized to east, central, and southern India, including where the CMC is located.

The Joint Clinical Research Center (JCRC) in Kampala, Uganda represents a clinical center of excellence with multiple local clinics across the country and in neighboring Kenya and South Africa. Since 1992, JCRC has been a pioneering site for implementation of HIV antiretroviral therapies in sub-Saharan Africa, and was a case study that inspired the U.S. President’s Emergency Plan for AIDS Relief (PEPFAR). Two-thirds of patients living with HIV and associated new infections are in the sub-Saharan African region [13]. Moreover, this region also has a high incidence of sickle cell trait carriers and the highest majority of newborns diagnosed with sickle cell disease in the world. The JCRC has apheresis capabilities and a transfusion exchange service for patients with sickle cell disease, as well as College of American Pathologists (CAP)-certified laboratories.

### Understanding the divide

Starting from the GGTI’s overarching goal of equitable access to gene therapies, and based on the collective experience of GGTI participants, four critical gap areas for gene therapy implementation in LMIC were identified: clinical readiness and implementation, new technology development, regulation and policy, and community outreach and education.

Clinical readiness and implementation relies on the capacity to perform all of the required interventions and quality tests for gene therapy to be administered, but to also be prepared for possible side effects and to manage supportive care, as well as to monitor patient outcomes. For example, a clinic evaluating gene therapy for sickle cell disease needs to be able to measure hemoglobin levels and variants, and resulting red blood cell function, as well as to support vaso-occlusive crisis intervention, and provide the care needed to get a patient within published clinical parameters for collection of blood stem cells to initiate and administer gene therapy [14, 15]. For each of these clinical activities there is a need for protocols, training, medications, diagnostics, reagents, monitoring equipment, and common data elements and platforms for real-time care monitoring and clinical intervention; access to each of these is often limited in LMIC.

Without established infrastructure for implementation of gene therapies in LMIC, place-of-care manufacturing provides an important solution to facilitate initial clinical trials. This requires the necessary equipment, reagents, materials and procedures to support manufacturing, as well as quality testing capabilities for release of gene-modified cellular products for clinical use. Knowledge transfer and training are key needs to support place-of-care manufacturing infrastructure and maintain good manufacturing practice for the long-term.

While some technologies exist that could support immediate clinical implementation in a place-of-care approach, many are not currently cost effective or sustainable in LMIC. Thus, new technologies must be developed in parallel that address the need to lower cost of goods and manufacturing times and to enable sustainable delivery of gene therapy in LMIC where regular patient access to centralized clinics is not always feasible.

All efforts to develop and engage LMIC in gene therapy trials must also reconcile different regulatory approval and reimbursement structures in these countries, which may or may not have appropriate regulatory approval pathways or reimbursement policies in place for genetic modification of human cells. In countries that do have a regulatory infrastructure in place, follow-up can be hit or miss. Thus, initial efforts must include a strong educational component that is sensitive and flexible to the priorities and needs of different governments and regulatory bodies, and to other stakeholders who will approve clinical implementation and any future commercialization efforts. In addition, the initiative should proactively engage with key stakeholders and contribute to development and implementation of a dynamic regulatory governance framework that anticipates systems needs for effective oversight of gene therapies today and in the future. The goal is to develop gene therapies which are affordable in LMIC, improving technological and logistical efficiencies that would enable progress towards sustainable reimbursement strategies in LMIC and non-LMIC alike.

Most importantly, community outreach to patients and people who could benefit from gene therapy must be our starting point. We must always provide the community with structured opportunities to inform when and how gene therapy will be implemented. Early and transparent communication including translation of gene therapy concepts into native languages with imagery that reflects the patients’ customs and ways of life is critical. The same concepts must be communicated to stakeholders who will impact gene therapy acceptance such as caregivers, as well as influential members of communities in which patients reside such as religious leaders. Understanding factors that make gene therapies acceptable in diverse settings will be critical, and this research must be adapted to local contexts. Integrating socio-behavioral scientists and bioethicists as part of clinical trial teams, particularly as trials are upscaled and require more participants, will be important to inform patient and participant-centered designs [16, 17]. Again, sensitivity and flexibility to the priorities and needs of communities and patients must drive gene therapy development to facilitate successful and sustainable adoption. Gene therapy introduction and access should be co-designed by participating communities if it is to be accepted, client-centered and sustained to avoid pitfalls other technologies have faced.

### A strategy to bridge the gap

Any approach ought to be developed in alignment with the therapeutic product profile (TPP) such as that described earlier this year for HIV cure [18]. Joint efforts with the Sunnylands Working Group and the HIV Cure Africa Acceleration Partnership are ongoing to develop a TPP for gene therapy-based approaches. Across the alliance, it was widely agreed that current state-of-the-art engineered lentiviral vectors for gene therapy of blood-related diseases should be the initial strategy pursued for entry trials in LMIC as these vectors have the most robust data available on safety and efficacy [19]. Based on current efforts in India, data from at least three patients treated with the proposed drug product in the U.S. or Europe will be required for approval of a clinical protocol to test locally. The drug product in gene therapy trials is generally considered defined by the cell type and lentiviral vector used for their genetic modification [20].

The source of patient cells and methods of administration into patient cells can vary considerably from study to study, but this does not change the regulatory definition of the drug product. Our initial efforts will focus upon use of devices, vectors and materials that have demonstrable safety and efficacy in place-of-care clinical trials in the U.S. and Europe [21, 22]. While place-of-care manufacturing approval for commercial gene-modified cells has not yet occurred in the U.S. or Europe, the lower cost and other benefits, particularly for resource-limited countries, are compelling. The use of local labor to manufacture the drug product and avoidance of custodial and logistical assurance costs associated with central manufacturing will improve affordability. Other advantages of place-of-care manufacturing include fresh-in, fresh-out product manufacturing, where fresh products have immediate therapeutic effects, rather than the delayed effects seen with cryopreserved products, simplified local product custody assurance and logistics, and reduced overall vein-to-vein manufacturing time. Furthermore, place-of-care manufacturing will facilitate stronger partnerships between innovators and clinicians, relationships which are key for long-term sustainability.

It is important to note that under current regulations in the U.S. and Europe, any use of a vector in a particular cell phenotype (i.e., the drug product), is subject to clinical trial holds if serious adverse events are reported, even when they occur in patients treated in a separate clinical trial with a significantly different manufacturing process on a different continent. For this reason, the GGTI is prioritizing non-commercial lentiviral vectors so as to minimize the risk of affecting active development programs and commercialization efforts [23, 24]. In addition, development of a competitive drug product that can be manufactured more cost effectively will bolster market competition, which in turn will inevitably drive down cost and improve patient access. At a high level, the GGTI emphasizes technologies that support more minimally manipulative ex vivo gene therapy approaches as well as potential future in vivo delivery strategies. The GGTI will continue involving investigators and institutions to develop novel technologies and processes under our shared vision.

To facilitate clinical readiness for patient diagnosis and monitoring, GGTI participants include researchers developing low-cost point-of-care technologies for diagnosing hemoglobinopathies and monitoring hemoglobin levels [25], red blood cell function [26, 27], and HIV viral loads [28]. Initial phase I clinical trials in the U.S. would include comparison of these low-cost, portable technologies with current state-of-the-art methods to support place-of-care approval and adoption in LMIC. To demonstrate that these combined technologies can facilitate expanded access to gene therapies, Phase I clinical trials will be implemented in multiple U.S. clinics that to date have not offered gene therapy trials to patients, under a multi-center trial approach led by an academic institution with experience in the successful conduct of blood cell gene therapy clinical trials. This approach aims to demonstrate that place-of-care manufacturing and testing can be accomplished in centers with limited or no prior gene therapy experience. Successful place-of-care manufacturing needs to demonstrate that the gene-modified cell product variation between each site is not significantly different than the expected variation at each site. Use of highly similar devices, materials and reagents for the generation and testing of gene-modified cell products will be essential for successful implementation of place-of-care cell manufacturing. This strategy will also permit clinicians and fellows from the CMC and JCRC to train alongside U.S. clinicians from these institutions under the auspices of an experienced clinical center. Centers in the U.S. have been identified, and discussions are underway.

While this strategy is designed to expand access and facilitate successful implementation in new clinics and countries, GGTI participants are simultaneously outlining LMIC regulatory processes and gaps, and developing relationships to facilitate governance education and guidance where appropriate. In the summer of 2020, the World Health Organization (WHO) led an effort to develop recommendations for harmonized regulatory frameworks for cell and gene therapy products so that regulators in LMIC have a path to bring such therapies forward and to determine their appropriateness, safety and efficacy. GGTI participants are working with the WHO to understand governance gaps at the CMC and JCRC, and to establish these sites as case studies in local implementation of regulatory governance. The WHO is the appropriate regulatory body to adjudicate where governance is not already in place for our LMIC of interest, India and Uganda, and country-based offices must make the request for WHO support in these efforts. Thus, GGTI participants are engaging key local stakeholders to facilitate establishment of case study sites and implementation of governance strategies, and to identify strategic governance that could be impacted by gene therapy initiatives, such as trade and import policies or agreements. The simultaneous engagement of both government bodies and the communities they serve is an important goal of the GGTI. Throughout this process GGTI will ensure that engagement with regulators in the two countries maintains a regulatory firewall (i.e., separation between developers and regulators), to promote effective oversight. Hence the role of the WHO’s local regulatory capacity is developed.

Foundational to these efforts is outreach and education. To facilitate community outreach and education, and to demonstrate our patient-centric mindset, the GGTI has identified individuals from the patient communities we hope to serve to participate in working group efforts. Currently, our International Community Advisory Board (iCAB) includes seven members, each of whom represent not only the patient communities living with sickle cell disease, beta-thalassemia and HIV, but also various aspects of other GGTI focus areas, including government relations, patient advocacy and technology development and implementation. We are striving to achieve representation from each participating country and are very close to this goal. Key efforts of the iCAB include developing resources to communicate a basic understanding, namely, what is gene therapy, why gene therapy is helpful, and how gene therapy is performed. iCAB members are facilitating translation into native languages and adapting appropriate imagery to facilitate community understanding of these concepts. In addition to the iCAB, GGTI will continuously seek ways to expand community involvement in this initiative, recognizing that community voices are heterogeneous and their representation needs change.

The GGTI is further adapting the same concepts for communication to stakeholders who are part of gene therapy regulatory approval and policy development processes, as well as influential community members. An important concept already identified includes the impact of cultural stigmatization experienced by patients living with sickle cell disease or HIV, which have some similarities but also distinct manifestations depending on local cultures. Early efforts contributed to the creation of an online resource for children diagnosed with sickle cell disease championed by the National Institutes of Health and developed by iCAB member, Olabimpe Olayiwola, in collaboration with the Cleveland Institute of Art’s Biomedical Art Program [29, 30]. Local efforts to engage patient communities in order to ascertain knowledge and gaps regarding gene therapy education were also employed. Indeed, grassroots efforts in Kampala by iCAB member, Moses Supercharger, were critical to development of the “three questions” approach (What is gene therapy? Why might gene therapy be helpful? How is gene therapy done?), and towards understanding the need for common language translation and flexibility in communication styles. For example, to encourage community adoption of antiretroviral drug therapies in the treatment of HIV infection, Supercharger utilizes his skills as an artist and television/radio presenter to engage the Ugandan community through song [https://bit.ly/3tgWBys]. Just as out-of-the-box technologies will be needed to physically implement gene therapies worldwide, our communication and outreach strategies to develop community participation must be equally flexible and creative.

Finally, GGTI is engaging researchers in the gene therapy field who have made initial proof-of-concept progress in various aspects of delivery, safety and efficacy. We plan to ensure that these leading pioneers have real-time access to the experience gained by LMIC implementation as described above.

### How we will get there

Just as the GGTI seeks to bring diverse stakeholders to the table, a diverse and flexible support approach is also key to the success of this initiative. Participation is voluntary. Multiple sponsors, including academic and nonprofit institutions that GGTI participants are affiliated with, are currently supporting this effort. While this grassroots funding strategy will be sufficient to launch the initiative, commercial participation and support will be key to long-term success. Through the efforts of the nonprofit organization Caring Cross [31], the GGTI is advancing a humanitarian licensing strategy to facilitate commercialization while supporting sustainable access in LMIC. Namely, licensing entities can establish market pricing in the top 15 most developed nations, as well as the European Market Countries, which in total represent 45 nations, but must offer best available pricing in LMIC if no other access considerations or limitations exist. The GGTI will also advocate for patient access programs and other mechanisms to increase affordability for patients in countries with market pricing. Moreover, the GGTI supports the empowerment of LMIC to develop novel technologies and processes that are patentable to facilitate entry into the gene therapy value chain, the most robust method for ensuring sustainability. To accomplish this, the GGTI will advocate for economic studies during the trials to support a business case for introducing gene therapies in LMIC early as a sustainable strategy for countries to enhance their paths to universal health care for improved population health outcomes and affordable health care for all.

Success of implementation will be measured as short- and long-term progress as follows: The first milestone will be successful opening of a phase I clinical trials to enrollment. This will be a critical goal for clinical implementation and regulation, as it will require the ability to reproducibly validate successful local cell product manufacture, where the manufacturing data is reviewed and approved by local regulatory authorities. Enrollment, treatment, and monitoring of patients in the clinical trial will be the next milestones where community outreach and engagement will be critical. Review of in-progress data by local regulatory agencies during initial clinical trials and expanded access will be an integral part of this process. Long-term goals include the implementation of next generation technologies and improved local operational efficiencies, with eventual full local regulatory approval of gene therapies in LMIC.

### The unpaved road ahead

Inevitably, multiple challenges must be met to achieve the successful implementation of gene therapies in LMIC. Starting with the hemoglobinopathies and HIV, diseases with disproportionate impact in LMIC, a pathway for clinical development and approval will be established that will be applicable to many other diseases, including those in oncology for which market approval for several products has already been attained in select developed countries. The efforts of the GGTI represent a path toward understanding the barriers to implement gene therapy in LMIC, and a commitment to engaging key stakeholders to find coherent and pragmatic solutions for these challenges so that, without exception, patients in need can have access to gene therapy no matter where they reside.
